# A New Power Topp–Leone Generated Family of Distributions with Applications

**DOI:** 10.3390/e21121177

**Published:** 2019-11-29

**Authors:** Rashad A. R. Bantan, Farrukh Jamal, Christophe Chesneau, Mohammed Elgarhy

**Affiliations:** 1 Department of Marine Geology, Faculty of Marine Sience, King AbdulAziz University, Jeddah 21551, Saudi Arabia; rbantan@kau.edu.sa; 2 Deanship of Scientific Research, King AbdulAziz University, Jeddah 21551, Saudi Arabia; 3 Department of Statistics, Govt. S.A Postgraduate College Dera Nawab Sahib, Bahawalpur, Punjab 63100, Pakistan; drfarrukh1982@gmail.com; 4 Department of Mathematics, Université de Caen, LMNO, Campus II, Science 3, 14032 Caen, France; 5 Valley High Institute for Management Finance and Information Systems, Obour, Qaliubia 11828, Egypt; m_elgarhy85@sva.edu.eg

**Keywords:** power Topp–Leone distribution, inverse exponential-G family, moments, entropy, estimation, data analysis, 60E05, 62E15, 62F10

## Abstract

In this paper, we introduce a new general family of distributions obtained by a subtle combination of two well-established families of distributions: the so-called power Topp–Leone-G and inverse exponential-G families. Its definition is centered around an original cumulative distribution function involving exponential and polynomial functions. Some desirable theoretical properties of the new family are discussed in full generality, with comprehensive results on stochastic ordering, quantile function and related measures, general moments and related measures, and the Shannon entropy. Then, a statistical parametric model is constructed from a special member of the family, defined with the use of the inverse Lomax distribution as the baseline distribution. The maximum likelihood method was applied to estimate the unknown model parameters. From the general theory of this method, the asymptotic confidence intervals of these parameters were deduced. A simulation study was conducted to evaluate the numerical behavior of the estimates we obtained. Finally, in order to highlight the practical perspectives of the new family, two real-life data sets were analyzed. All the measures considered are favorable to the new model in comparison to four serious competitors.

## 1. Introduction

Owing to the growing amount of data from various applied fields and unstoppable computer progress, there is increasing motivation on developing efficient and flexible statistical models. Such models can be derived from general families of distributions having desirable properties, such as those constructed from a generator distribution. The main idea of this construction is to add shape parameter(s) to a baseline distribution with the aim to upgrade its flexibility level. Among the well-known examples of such families, there are the beta-G [1], Kumaraswamy-G [2], Weibull-GG [3], Garhy-G [4], type II half logistic-G [5], Transmuted Topp–Leone G [6], generalized odd log-logistic-G [7], odd Fréchet-G [8], power Lindley-G [9], Fréchet Topp–Leone-G [10], exponentiated generalized Topp–Leone-G [11], and truncated inverted Kumaraswamy-G [12]. We also refer to the exhaustive survey in [13]. Recently, several researchers used the Topp–Leone (TL) distribution as generator distribution to develop new general families, reaching the aims of simplicity and flexibility. Among them, Ref. [14] proposed the Topp–Leone-G (TL-G) family, Ref. [15] introduced the power TL-G (PTL-G) family, Ref. [16] introduced the generalized TL-G family, Ref. [17] studied the type II TL-G family, and [18] proposed the type II generalized TL-G family.

For the purposes of this paper, let us describe in detail the PTL-G family from [15]. The PTL-G family is defined by the following cumulative distribution function (cdf):

$$F\left(x;\alpha ,\beta ,\xi \right)=G{\left(x;\xi \right)}^{\alpha \beta }{\left[2-G{\left(x;\xi \right)}^{\beta }\right]}^{\alpha },\;x\in R,$$

with α,β>0, where G(x;ξ) is a cdf of a baseline continuous distribution which may depend on a vector parameter ξ; i.e., $\xi =(\xi_{1},\xi_{2},\ldots )$. As indicated by the name, the construction of the family uses the so-called power Topp–Leone distribution as the generator distribution. In comparison to the (power one) TL-G family, Ref. [15] demonstrated the significant impact of the parameter β on the shapes of the probability density and hazard rate functions, providing desirable modeling properties. This is particularly flagrant with the consideration of the gamma distribution as the baseline distribution, as illustrated by the graphics and applications of [15].

In a parallel work, beyond the TL distribution and its extensions, Ref. [19] introduced the inverse exponential-G (IE-G) family, based on the inverse exponential distribution as the generator distribution, and defined by the following cdf:

$$F\left(x;\xi \right)=e^{1-\frac{1}{G(x;\xi )}},\;x\in R.$$

The main features of this family are being simple, with no new, additional parameters, and having a completely different nature of the former baseline cdf G(x;ξ) owing to the combination the exponential (implicit) odd functions. An immediate remark illustrating this claim is the following: it has a fastest rate of decay to 0 when G(x;ξ)→0. By the consideration of a practical data set and the exponential distribution as baseline distribution, Ref. [19] shows that the corresponding model is better than the Lindley and exponential models (all having the same number of parameters). The nice results behind the IE-G family have been the driver for more investigations, with extended or modified versions of this family. We refer the reader to [8,20] for the odd Fréchet-G family, Ref. [21] for the extended odd Fréchet-G family, and [22] for the modified odd Fréchet-G family.

In this paper, in view of the previously mentioned literature, we introduce a new family of distributions by combining, in some senses, the PTL-G and IE-G families. It is defined by composition of their respective cdfs, i.e., by the cdf given by

(1) $$\begin{array}{c} F\left(x;\alpha ,\beta ,\xi \right)=e^{\alpha \beta \left(1-\frac{1}{G(x;\xi )}\right)}{\left[2-e^{\beta \left(1-\frac{1}{G(x;\xi )}\right)}\right]}^{\alpha },\;x\in R, \end{array}$$

with α,β>0. Thus, this cdf can be view as a polyno-exponential transformation of the baseline cdf G(x;ξ). The new family is called the new power TL-G (NPTL-G) family. Thus, by construction, we aim to combine the benefits of the PTL-G and IE-G families, and thus, create new statistical perspectives of various kinds. The key motivations behind the NPTL-G family are the following.

1. To provide very simple models and create new simple distributions.
2. To improve the flexibility of existing distributions on various aspects (such as mode, median, skewness, and kurtosis…).
3. To provide better fits than competing modified models having the same of higher number of parameters.

We support these claims both in full generality and by putting the light on the special member of the NPTL-G family defined with the inverse Lomax (ILx) distribution as the baseline distribution (the reason of this choice will be explained later). The resulting distribution, called the new power Topp–Leone inverse Lomax (NPTLILx) distribution, offers a new three-parameter lifetime distribution, with a high potential of applicability. We illustrate that by the means of two practical data sets with different features: the first one is from [23] and is about active repair times for airborne communication transceiver, and the second one is from [24] and is about actual tax revenue in Egypt. Favorable results were obtained for the proposed model in comparison to serious competitors, motivating its use wider statistical uses.

The contents of this paper are organized as follows. In Section 2, the basics of the NPTL-G family are presented, as is the NPTLILx distribution. Various mathematical properties of the family are discussed in Section 3. Section 4 is devoted to the estimation of the unknown parameters from the NPTLILx model, with a comprehensive simulation study. The data analyses are shown in Section 5 with numerical and graphical illustrations. A conclusion and perspectives are formulated in Section 6.

## 2. Basics of the NPTL-G Family

The basics of the NPTL-G family are presented in this section, with a focus on the main functions of interest.

### 2.1. Probability Density Function

Upon differentiation of F(x;α,β,ξ) according to *x*, owing to (1), the probability density function (pdf) of the NPTL-G family is given by

(2) $$\begin{array}{c} f\left(x;\alpha ,\beta ,\xi \right)=2\alpha \beta \frac{g(x;\xi )}{G{\left(x;\xi \right)}^{2}}e^{\alpha \beta \left(1-\frac{1}{G(x;\xi )}\right)}\left[1-e^{\beta \left(1-\frac{1}{G(x;\xi )}\right)}\right]{\left[2-e^{\beta \left(1-\frac{1}{G(x;\xi )}\right)}\right]}^{\alpha -1},\;x\in R, \end{array}$$

where g(x;ξ) is the probability density function corresponding to G(x;ξ). From this expression, some asymptotic results on f(x;α,β,ξ) can be derived. When G(x;ξ)→0, we have

$$f\left(x;\alpha ,\beta ,\xi \right)∼2\alpha \beta \frac{g(x;\xi )}{G{\left(x;\xi \right)}^{2}}e^{\alpha \beta \left(1-\frac{1}{G(x;\xi )}\right)}.$$

Furthermore, when G(x;ξ)→1, we have

$$f\left(x;\alpha ,\beta ,\xi \right)∼2\alpha \beta^{2}g\left(x;\xi \right)\left[1-G(x;\xi )\right].$$

The variations of f(x;α,β,ξ) can be studied in a standard manner, starting with the critical point(s) given by the solution of the non-linear equation according to *x*: ${\left\{\ln[f(x;\alpha ,\beta ,\xi )]\right\}}^{\prime }=0$, with

$$\begin{array}{cc} {\left\{\ln[f(x;\alpha ,\beta ,\xi )]\right\}}^{\prime } & =\frac{g{\left(x;\xi \right)}^{\prime }}{g(x;\xi )}-2\frac{g(x;\xi )}{G(x;\xi )}+\alpha \beta \frac{g(x;\xi )}{G{\left(x;\xi \right)}^{2}}-\beta \frac{g\left(x;\xi \right)e^{\beta \left(1-\frac{1}{G(x;\xi )}\right)}}{G{\left(x;\xi \right)}^{2}\left[1-e^{\beta \left(1-\frac{1}{G(x;\xi )}\right)}\right]}\\ \; & -\beta \left(\alpha -1\right)\frac{g\left(x;\xi \right)e^{\beta \left(1-\frac{1}{G(x;\xi )}\right)}}{G{\left(x;\xi \right)}^{2}\left[2-e^{\beta \left(1-\frac{1}{G(x;\xi )}\right)}\right]}. \end{array}$$

Then, for a critical point $x_{c}$, the sign of ${\left\{\ln[f(x;\alpha ,\beta ,\xi )]\right\}}^{\prime \prime }{|}_{x=x_{c}}$ is informative on its nature (minimum, maximum, or inflection point).

### 2.2. Hazard Rate Function

The hazard rate function (hrf) of the NPTL-G family is given by

$$\begin{array}{cc} h(x;\alpha ,\beta ,\xi ) & =\frac{f(x;\alpha ,\beta ,\xi )}{1-F(x;\alpha ,\beta ,\xi )}\\ \; & =\frac{2\alpha \beta g\left(x;\xi \right)e^{\alpha \beta \left(1-\frac{1}{G(x;\xi )}\right)}\left[1-e^{\beta \left(1-\frac{1}{G(x;\xi )}\right)}\right]{\left[2-e^{\beta \left(1-\frac{1}{G(x;\xi )}\right)}\right]}^{\alpha -1}}{G{\left(x;\xi \right)}^{2}\left\{1-e^{\alpha \beta \left(1-\frac{1}{G(x;\xi )}\right)}{\left[2-e^{\beta \left(1-\frac{1}{G(x;\xi )}\right)}\right]}^{\alpha }\right\}},\;x\in R. \end{array}$$

Some asymptotic results on h(x;α,β,ξ) are presented below. When G(x;ξ)→0, we have

$$h\left(x;\alpha ,\beta ,\xi \right)∼2\alpha \beta \frac{g(x;\xi )}{G{\left(x;\xi \right)}^{2}}e^{\alpha \beta \left(1-\frac{1}{G(x;\xi )}\right)}.$$

Additionally, when G(x;ξ)→1, we have

$$h\left(x;\alpha ,\beta ,\xi \right)∼2g\left(x;\xi \right){\left[1-G(x;\xi )\right]}^{-1}.$$

Thus, the parameters α and β have a significant effect on the asymptotes when G(x;ξ)→0, but no effect when G(x;ξ)→1. The variations of h(x;α,β,ξ) can be studied in similar manner to f(x;α,β,ξ) by using the relation ${\left\{\ln[h(x;\alpha ,\beta ,\xi )]\right\}}^{\prime }={\left\{\ln[f(x;\alpha ,\beta ,\xi )]\right\}}^{\prime }+h\left(x;\alpha ,\beta ,\xi \right)$.

### 2.3. A Special Member: The NPTLILx Distribution

The NPTL-G family contains distributions of various natures, depending on the choice of the baseline distribution. In this study, as evoked in the introduction, we chose the inverse Lomax distribution with shape parameter θ>0 as the baseline distribution to define the NPTLILx distribution. Thus, it is defined by the following cdf:

$$G\left(x;\theta \right)={\left(1+x^{-1}\right)}^{-\theta },\;x>0,$$

(another parameter of the former definition of the inverse Lomax distribution has been reduced to 1 for the purposes of the paper). Let us now briefly motivate this choice. As suggested by its name, the inverse Lomax distribution is the distribution of the random variable Y=1/X, where *X* denotes a random variable following the standard Lomax distribution (with parameters θ and 1). The corresponding pdf and hrf are, respectively, given by

$$g\left(x;\theta \right)=\theta x^{-2}{\left(1+x^{-1}\right)}^{-\theta -1}$$

and

$$h\left(x;\theta \right)=\frac{\theta x^{-2}{\left(1+x^{-1}\right)}^{-\theta -1}}{1-{\left(1+x^{-1}\right)}^{-\theta }},\;x>0.$$

In addition to being simple, it has been proven to be a very flexible to model data having a subjacent non-monotonic hrf. Further details and applications can be found in [25,26,27].

Thus, the NPTLILx distribution is defined by the following cdf:

(3) $$\begin{array}{c} F\left(x;\alpha ,\beta ,\theta \right)=e^{\alpha \beta \left[1-{\left(1+x^{-1}\right)}^{\theta }\right]}{\left\{2-e^{\beta \left[1-{\left(1+x^{-1}\right)}^{\theta }\right]}\right\}}^{\alpha },\;x>0, \end{array}$$

with α,β,θ>0. The corresponding pdf and hrf are given by, respectively,

(4) $$\begin{array}{c} f(x;\alpha ,\beta ,\theta )=\\ 2\alpha \beta \theta x^{-2}{\left(1+x^{-1}\right)}^{\theta -1}e^{\alpha \beta \left[1-{\left(1+x^{-1}\right)}^{\theta }\right]}\left\{1-e^{\beta \left[1-{\left(1+x^{-1}\right)}^{\theta }\right]}\right\}{\left\{2-e^{\beta \left[1-{\left(1+x^{-1}\right)}^{\theta }\right]}\right\}}^{\alpha -1} \end{array}$$

and

$$\begin{array}{c} h(x;\alpha ,\beta ,\theta )=\\ \frac{2\alpha \beta \theta x^{-2}{\left(1+x^{-1}\right)}^{\theta -1}e^{\alpha \beta \left[1-{\left(1+x^{-1}\right)}^{\theta }\right]}\left\{1-e^{\beta \left[1-{\left(1+x^{-1}\right)}^{\theta }\right]}\right\}{\left\{2-e^{\beta \left[1-{\left(1+x^{-1}\right)}^{\theta }\right]}\right\}}^{\alpha -1}}{1-e^{\alpha \beta \left[1-{\left(1+x^{-1}\right)}^{\theta }\right]}{\left\{2-e^{\beta \left[1-{\left(1+x^{-1}\right)}^{\theta }\right]}\right\}}^{\alpha }},\;x>0. \end{array}$$

**Figure 1:**
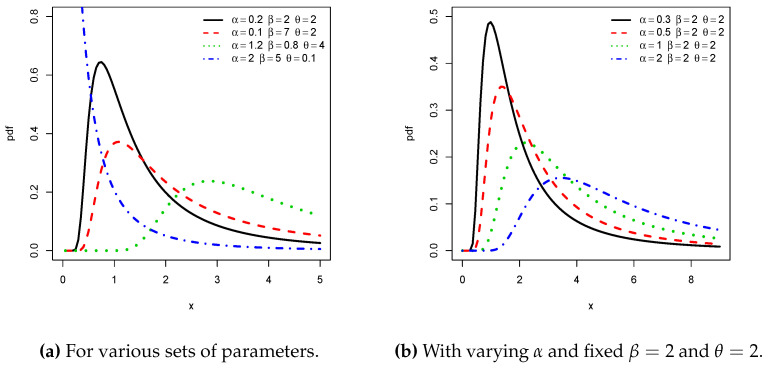
Plots of some probability density functions (pdfs) of the new power Topp–Leone inverse Lomax (NPTLILx) distribution.

**Figure 2:**
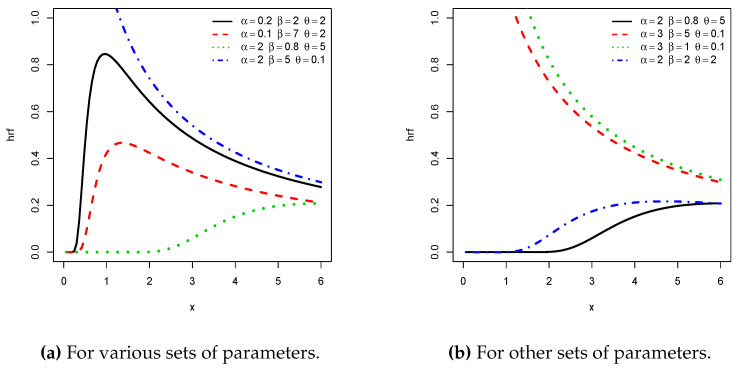
Plots of some hazard rate functions (hrfs) of the NPTLILx distribution.

Possible shapes of the pdf and hrf of the NPTLILx distribution are illustrated in Figure 1 and Figure 2, respectively. In particular, from Figure 1, we see that the pdf can be right skewed and reversed-J shaped. From Figure 2, we see that the hrf can be increasing, decreasing, upside down, and bathtub shaped. All these curvature properties are known to be desirable to create flexible statistical models.

## 3. Some Mathematical Properties

The section presents some important mathematical properties of the NPTL-G family.

### 3.1. On a Stochastic Ordering

The following result shows some inequalities involving F(x;α,β,ξ).

**Proposition** **1.** *For any x∈R such that G(x;ξ)>0, the following inequalities hold:*

$$e^{\alpha \beta \left(1-\frac{1}{G(x;\xi )}\right)}{\left[2-G{\left(x;\xi \right)}^{\beta }\right]}^{\alpha }\leq F\left(x;\alpha ,\beta ,\xi \right)\leq 2^{\alpha }e^{\alpha \beta \left(1-\frac{1}{G(x;\xi )}\right)}.$$

**Proof.**  The bracket term in the definition of F(x;α,β,ξ) given by (1) is central. Since $e^{\beta \left(1-\frac{1}{G(x;\xi )}\right)}\in \left(0,1\right)$, we have $2-e^{\beta \left(1-\frac{1}{G(x;\xi )}\right)}\leq 2$, implying the second inequality. For the first inequality, the following well-known logarithmic inequality: for y>−1, $\frac{y}{1+y}\leq \ln\left(1+y\right)$ gives $e^{1-\frac{1}{1+y}}\leq 1+y$, implying that $e^{1-\frac{1}{G(x;\xi )}}\leq G\left(x;\xi \right)$ by taking y=G(x;ξ)−1. Therefore, we have $e^{\beta \left(1-\frac{1}{G(x;\xi )}\right)}\leq G{\left(x;\xi \right)}^{\beta }$, and a fortiori, $2-G{\left(x;\xi \right)}^{\beta }\leq 2-e^{\beta \left(1-\frac{1}{G(x;\xi )}\right)}$. The first inequality follows. This ends the proof of Proposition 1. □

An immediate consequence of Proposition 1 is the following stochastic ordering result:

$$F_{*}\left(x;\alpha ,\beta ,\xi \right)\leq F\left(x;\alpha ,\beta ,\xi \right),$$

where $F_{*}\left(x;\alpha ,\beta ,\xi \right)=e^{\alpha \beta \left(1-\frac{1}{G(x;\xi )}\right)}$ is the cdf of the exponentiated IE-G family (with power parameter αβ).

Another stochastic ordering result comes from the following remark: the function $F_{o}\left(x;\alpha ,\beta ,\xi \right)$ given by

$$F_{o}\left(x;\alpha ,\beta ,\xi \right)=e^{\alpha \beta \left(1-\frac{1}{G(x;\xi )}\right)}{\left[2-G{\left(x;\xi \right)}^{\beta }\right]}^{\alpha },\;x\in R,$$

has the properties of a cdf, with the corresponding pdf given by

$$f_{o}\left(x;\alpha ,\beta ,\xi \right)=\alpha \beta \frac{g(x;\xi )}{G{\left(x;\xi \right)}^{2}}e^{\alpha \beta \left(1-\frac{1}{G(x;\xi )}\right)}\left\{2-\left[1+G\left(x;\xi \right)\right]G{\left(x;\xi \right)}^{\beta }\right\}{\left[2-G{\left(x;\xi \right)}^{\beta }\right]}^{\alpha -1},\;x\in R.$$

To the best of our knowledge, it is new in the literature (and out the scope of this paper).

### 3.2. Quantile Function with Some Related Measures and Functions

The quantile function (qf) of the NPTL-G family is expressed in the following result.

**Proposition** **2.** *The qf of the NPTL-G family is given by*

$$Q\left(u;\alpha ,\beta ,\xi \right)=Q_{G}\left[{\left\{1-\frac{1}{\beta }\ln\left(1-\sqrt{1-u^{\frac{1}{\alpha }}}\right)\right\}}^{-1};\xi \right],\;u\in \left(0,1\right),$$

*where $Q_{G}\left(u;\xi \right)$ is the qf corresponding to G(x;ξ).*

**Proof.**  For the sake of simplicity, let us set $x_{u}=Q\left(u;\alpha ,\beta ,\xi \right)$ for u∈(0,1). Then, by the definition of a qf, $x_{u}$ satisfies the non-linear equation: $u=F(x_{u};\alpha ,\beta ,\xi )$, implying that $u=e^{\alpha \beta \left(1-\frac{1}{G(x_{u};\xi )}\right)}{\left[2-e^{\beta \left(1-\frac{1}{G(x_{u};\xi )}\right)}\right]}^{\alpha }$; hence, $u^{\frac{1}{\alpha }}=e^{\beta \left(1-\frac{1}{G(x_{u};\xi )}\right)}\left[2-e^{\beta \left(1-\frac{1}{G(x_{u};\xi )}\right)}\right]$, which is equivalent to solving the polynomial equation according to *y*: $y^{2}-2y+u^{\frac{1}{\alpha }}=0$, with $y=e^{\beta \left(1-\frac{1}{G(x_{u};\xi )}\right)}$. By determining the two roots of this polynomial, keeping only the one in the unit interval (since y∈(0,1)), we get $y=1-\sqrt{1-u^{\frac{1}{\alpha }}}$. After some algebra, we get $G\left(x_{u};\xi \right)={\left\{1-\frac{1}{\beta }\ln\left(1-\sqrt{1-u^{\frac{1}{\alpha }}}\right)\right\}}^{-1}$. The desired result follows by compounding with $Q_{G}\left(u;\xi \right)$, ending the proof of Proposition 2. □

From the qf, we can define several quantities of importance, providing distributional properties of the family. Some of them are presented below.

The three quartiles of the NPTL-G family are defined by $Q_{1}=Q\left(1/4;\alpha ,\beta ,\xi \right)$, $Q_{2}=Q\left(1/2;\alpha ,\beta ,\xi \right)$, and $Q_{3}=Q\left(3/4;\alpha ,\beta ,\xi \right)$. In particular, the median of the NPTL-G family is given by

$$M=Q_{2}=Q_{G}\left[{\left\{1-\frac{1}{\beta }\ln\left(1-\sqrt{1-{\left(\frac{1}{2}\right)}^{\frac{1}{\alpha }}}\right)\right\}}^{-1};\xi \right].$$

Additionally, the inter-quartile range is given by $IQR=Q_{3}-Q_{1}$, allowing one to define the Galton coefficient of skewness and the Moors coefficient of kurtosis, given by, respectively,

$$S=\frac{Q_{3}+Q_{1}-2M}{IQR}$$

and

$$K=\frac{Q(7/8;\alpha ,\beta ,\xi )-Q(5/8;\alpha ,\beta ,\xi )+Q(3/8;\alpha ,\beta ,\xi )-Q(1/8;\alpha ,\beta ,\xi )}{IQR}.$$

See [28,29] for more details on these coefficients, respectively.

On the other hand, upon differentiation of Q(u;α,β,ξ) according to *u*, the corresponding quantile density function is given by

$$\begin{array}{cc} q(u;\alpha ,\beta ,\xi ) & =\frac{u^{\frac{1}{\alpha }-1}}{2\alpha \beta \sqrt{1-u^{1/\alpha }}\left(1-\sqrt{1-u^{\frac{1}{\alpha }}}\right){\left\{1-\frac{1}{\beta }\ln\left(1-\sqrt{1-u^{\frac{1}{\alpha }}}\right)\right\}}^{2}}\times \\ \; & q_{G}\left[{\left\{1-\frac{1}{\beta }\ln\left(1-\sqrt{1-u^{\frac{1}{\alpha }}}\right)\right\}}^{-1};\xi \right],\;u\in \left(0,1\right), \end{array}$$

where $q_{G}\left(u;\xi \right)$ is the quantile density function corresponding to G(x;ξ). Also, the hazard quantile function is defined by

$$\begin{array}{cc} H(u;\alpha ,\beta ,\xi ) & =\frac{1}{\left(1-u)q(u;\alpha ,\beta ,\xi \right)}\\ \; & =\frac{2\alpha \beta \sqrt{1-u^{1/\alpha }}\left(1-\sqrt{1-u^{\frac{1}{\alpha }}}\right){\left\{1-\frac{1}{\beta }\ln\left(1-\sqrt{1-u^{\frac{1}{\alpha }}}\right)\right\}}^{2}}{\left(1-u\right)u^{\frac{1}{\alpha }-1}}\times \\ \; & {\left\{q_{G}\left[{\left\{1-\frac{1}{\beta }\ln\left(1-\sqrt{1-u^{\frac{1}{\alpha }}}\right)\right\}}^{-1};\xi \right]\right\}}^{-1},\;u\in \left(0,1\right). \end{array}$$

These functions have central roles in reliability. Further details can be found in [30].

Last but not least, the qf allows us to generate values from members of the NPTL-G family. This property will be used in Section 4.2 in the context of the NPTLILx distribution; i.e., with the qf given by $Q_{G}\left(u;\theta \right)={\left(u^{-\frac{1}{\theta }}-1\right)}^{-1}$, u∈(0,1), so

$$Q\left(u;\alpha ,\beta ,\theta \right)={\left({\left\{1-\frac{1}{\beta }\ln\left(1-\sqrt{1-u^{\frac{1}{\alpha }}}\right)\right\}}^{\frac{1}{\theta }}-1\right)}^{-1},\;u\in \left(0,1\right).$$

As a numerical illustration, Table 1 shows the values of $Q_{1}$, *M*, $Q_{3}$, *S*, and *K* of the NPTLILx distribution for some parameter values.

We see in Table 1 that the effects of α, β, and θ on the quartiles are significant (we always have S>0 so the distribution is right-skewed and moderate variations for *K*).

### 3.3. Series Expansion

The exp-G family of distributions, introduced by [31], is defined by the following cdf: $G_{\gamma }\left(x;\xi \right)=G{\left(x;\xi \right)}^{\gamma }$, x∈R, with γ>0. The corresponding pdf is given by

$$g_{\gamma }\left(x;\xi \right)=\gamma g\left(x;\xi \right)G_{\gamma -1}\left(x;\xi \right),\;x\in R.$$

The interesting part of the exp-G family is to have well-known properties for a lot of baseline cdfs G(x;ξ). For instance, the member of the exp-G family defined with the inverse Lomax distribution as baseline with shape parameter θ becomes the inverse Lomax distribution with shape parameter γθ.

The following result concerns a series expansion for the pdf of the NPTL-G family in terms of pdfs of the exp-G family.

**Proposition** **3.** *We have the following series expansion:*

$$\begin{array}{c} f\left(x;\alpha ,\beta ,\xi \right)=\sum_{k,\ell ,m=0}^{+\infty }\sum_{q=1}^{m+\ell }\omega_{k,\ell ,m,q}g_{q}\left(x;\xi \right), \end{array}$$

*where*

ωk,ℓ,m,q=αk−ℓmm+ℓq2α−k1ℓ!(α+k)ℓβℓ(−1)k+ℓ+m+q,

*with the notation: ba=b(b−1)…(b−a+1)/a!.*

**Proof.**  We first investigate a series expansion of F(x;α,β,ξ) based on the Equation (1). Since $e^{\beta \left(1-\frac{1}{G(x;\xi )}\right)}/2\in \left(0,1\right)$, the generalized binomial formula gives

2−eβ1−1G(x;ξ)α=∑k=0+∞αk2α−k(−1)kekβ1−1G(x;ξ).

On the other hand, thanks to the power series of the exponential function, we get

$$\begin{array}{c} e^{\left(\alpha +k\right)\beta \left(1-\frac{1}{G(x;\xi )}\right)}=\sum_{\ell =0}^{+\infty }\frac{1}{\ell !}{\left(\alpha +k\right)}^{\ell }\beta^{\ell }{\left(1-\frac{1}{G(x;\xi )}\right)}^{\ell }. \end{array}$$

Now, it follows from the generalized and standard binomial formulas that

1−1G(x;ξ)ℓ=∑m=0+∞−ℓm(−1)ℓ+m[1−G(x;ξ)]m+ℓ=∑m=0+∞∑q=0m+ℓ−ℓmm+ℓq(−1)ℓ+m+qGq(x;ξ).

By combining all the above equalities together, we obtain

$$\begin{array}{c} F\left(x;\alpha ,\beta ,\xi \right)=\sum_{k,\ell ,m=0}^{+\infty }\sum_{q=0}^{m+\ell }\omega_{k,\ell ,m,q}G_{q}\left(x;\xi \right). \end{array}$$

Upon differentiation of F(x;α,β,ξ) according to *x*, we get the desired result, by removing the term in q=0, which vanished. Proposition 3 is proven. □

### 3.4. General Moments with Some Related Measures and Functions

Let *X* be a random variable having the cdf given by (1) (defined on a probability space (Ω,A,P), with an expectation denoted by *E*). Then, for any function ϕ(x) (such that all the following introduced quantities exist or converge), we have

$$\begin{array}{cc} E\left[\phi (X)\right] & =\int_{-\infty }^{+\infty }\phi \left(x\right)f\left(x;\alpha ,\beta ,\xi \right)dx\\ \; & =\int_{-\infty }^{+\infty }\phi \left(x\right)2\alpha \beta \frac{g(x;\xi )}{G{\left(x;\xi \right)}^{2}}e^{\alpha \beta \left(1-\frac{1}{G(x;\xi )}\right)}\left[1-e^{\beta \left(1-\frac{1}{G(x;\xi )}\right)}\right]{\left[2-e^{\beta \left(1-\frac{1}{G(x;\xi )}\right)}\right]}^{\alpha -1}dx. \end{array}$$

Two equivalent expressions involving already introduced qfs are as follows:

$$\begin{array}{c} E\left[\phi (X)\right]=\int_{0}^{1}\phi \left[Q_{G}\left(u;\xi \right)\right]2\alpha \beta u^{-2}e^{\alpha \beta \left(1-\frac{1}{u}\right)}\left[1-e^{\beta \left(1-\frac{1}{u}\right)}\right]{\left[2-e^{\beta \left(1-\frac{1}{u}\right)}\right]}^{\alpha -1}du \end{array}$$

and

$$\begin{array}{c} E\left[\phi (X)\right]=\int_{0}^{1}\phi \left\{Q_{G}\left[{\left\{1-\frac{1}{\beta }\ln\left(1-\sqrt{1-u^{\frac{1}{\alpha }}}\right)\right\}}^{-1};\xi \right]\right\}du. \end{array}$$

Numerical solutions exist to evaluate them for given G(x;ξ), ϕ(x) and α, β, and θ. Alternatively, we can consider Proposition 3, which implies that

(5) $$\begin{array}{c} E\left[\phi (X)\right]=\sum_{k,\ell ,m=0}^{+\infty }\sum_{q=1}^{m+\ell }\omega_{k,\ell ,m,q}U_{q}\left(\phi ,G\right), \end{array}$$

where

$$U_{q}\left(\phi ,G\right)=\int_{-\infty }^{+\infty }\phi \left(x\right)g_{q}\left(x;\xi \right)dx=q\int_{0}^{1}u^{q-1}\phi \left[Q_{G}\left(u;\xi \right)\right]du.$$

In some circumstances, truncated sums can be considered for practical purposes; for a large integer *K*, the following approximation reveals to be tractable and efficient:

$$\begin{array}{c} E\left[\phi (X)\right]\approx \sum_{k,\ell ,m=0}^{K}\sum_{q=1}^{m+\ell }\omega_{k,\ell ,m,q}U_{q}\left(\phi ,G\right). \end{array}$$

Some specific choices for ϕ(x) are of particular interest. Some of them are discussed below.

- By taking $\phi \left(x\right)=x^{s}$, we get the *s*-th moment of *X*—i.e., $\mu_{s}^{\prime }=E\left(X^{s}\right)$, including the mean of *X*, i.e., $\mu =\mu_{1}^{\prime }=E\left(X\right)$—and allow the expression the variance of *X*; i.e., $\sigma^{2}=\mu_{2}^{\prime }-{\left(\mu_{1}^{\prime }\right)}^{2}$.
- By taking $\phi \left(x\right)={\left(x-\mu \right)}^{s}$, we get the *s*-th central moment of *X*, i.e., $\mu_{s}=E\left[{\left(X-\mu \right)}^{s}\right]$, allowing one to calculate the *s*-th general coefficient of *X* given by $C_{s}=\mu_{s}/\sigma^{s}$, among others. This coefficient is useful to investigate the skewness and kurtosis properties of *X*.
- By taking $\phi \left(x\right)=e^{tx}$, we get the moment generation function of *X* according to the variable *t*; i.e., $M\left(t\right)=E\left(e^{tX}\right)$. It is well-known that $\mu_{s}^{\prime }=M{\left(t\right)}^{\left(s\right)}{|}_{t=0}$.
- By taking $\phi \left(x\right)=e^{itx}$, we get the characteristic function of *X* according to the variable *t*; i.e., $\varphi \left(t\right)=E\left(e^{itX}\right)$. In a same title of the cdf, the characteristic function entirely determines the NPTL-G family.
- By taking $\phi \left(x\right)=\phi_{y}\left(x\right)=x^{s}1_{\left\{x\leq y\right\}}$, which is equal to $x^{s}$ if x≤y and 0 otherwise, we get the *s*-th incomplete moment of *X* according to the variable *y*; i.e., $\mu_{s}^{\prime }\left(y\right)=E\left(X^{s}1_{\left\{X\leq y\right\}}\right)$. This function is useful to define mean deviations of *X*, the corresponding residual life function, Bonferroni and Lorenz curves, and others.

In the case of the NPTLILx distribution, since $f\left(x;\alpha ,\beta ,\theta \right)∼2\alpha \beta^{2}\theta^{2}x^{-3}$ when x→+∞, the mean exists but the variance does not exist, nor do moments of order greater to 2 (there is no problem when x→0). However, all the incomplete moments exist for any fixed y>0. In this regard, Table 2 provides the four first incomplete moments for *X* with y=1000.

### 3.5. Shannon Entropy

Here, we study the Shannon entropy of the NPTL-G family as defined by [32]. We recall that the Shannon entropy of a random variable measures the amount of uncertainty for the outcome of this variable. A high entropy reveals a high degree of uncertainty.

Now, let *X* be a random variable having the cdf given by (1). Then, the Shannon entropy of *X* is defined by

$$\begin{array}{c} \eta =-E\{\ln\left[f\left(X;\alpha ,\beta ,\xi \right)\right\}=-\int_{-\infty }^{+\infty }\ln\left[f\left(x;\alpha ,\beta ,\xi \right)\right]f\left(x;\alpha ,\beta ,\xi \right)dx. \end{array}$$

By the use of any mathematical software, for a given baseline cdf G(x;ξ), ϕ(x) and α, β, and θ, we can determine this integral. Another approach consists of developing η by the use of the pdf given by (2):

$$\begin{array}{cc} \eta & =-\ln\left(2\right)-\ln\left(\alpha \right)-\ln\left(\beta \right)-\alpha \beta -E\left\{\ln[g(X;\xi )]\right\}+2E\left\{\ln[G(X;\xi )]\right\}+\alpha \beta E\left[\frac{1}{G(X;\xi )}\right]\\ \; & -E\left\{\ln\left[1-e^{\beta \left(1-\frac{1}{G(X;\xi )}\right)}\right]\right\}-\left(\alpha -1\right)E\left\{\ln\left[2-e^{\beta \left(1-\frac{1}{G(X;\xi )}\right)}\right]\right\}. \end{array}$$

Some expectation terms can be expressed by using (5) with an appropriate function ϕ(x) as soon as $U_{q}\left(\phi ,G\right)$ exists and the sums converge.

In the context of the NPTLILx distribution, some values of η are collected in Table 3 for some parameter values.

In Table 3, the values belongs to the wide interval [−12.8,3.55], meaning that α, β, and θ have an important impact on the amount of information quantified by η.

## 4. Estimation with Numerical Results

In this section, we investigate the NPTLILx model characterized by the cdf given by (3). Thanks to its attractive theoretical and practical properties, the maximum likelihood method is used to estimate the parameters α, β, and θ. Numerical results attest to the efficiency of the estimates obtained.

Hereafter, we consider a random variable *X* following the NPTLILx distribution with parameters α, β, and θ.

### 4.1. Maximum Likelihood Estimation

Let $x_{1},\ldots ,x_{n}$ be a random sample of size *n* of *X*. Then, by using the pdf given by (4), the likelihood and log-likelihood functions are, respectively, given by

$$\begin{array}{cc} \; & L\left(\alpha ,\beta ,\theta \right)=\prod_{i=1}^{n}f\left(x_{i};\alpha ,\beta ,\theta \right)\\ \; & ={\left(2\alpha \beta \theta \right)}^{n}\prod_{i=1}^{n}x_{i}^{-2}{\left(1+x_{i}^{-1}\right)}^{\theta -1}e^{\alpha \beta \left[1-{\left(1+x_{i}^{-1}\right)}^{\theta }\right]}\left\{1-e^{\beta \left[1-{\left(1+x_{i}^{-1}\right)}^{\theta }\right]}\right\}{\left\{2-e^{\beta \left[1-{\left(1+x_{i}^{-1}\right)}^{\theta }\right]}\right\}}^{\alpha -1} \end{array}$$

and

$$\begin{array}{cc} \ell (\alpha ,\beta ,\theta ) & =\ln\left[L\left(\alpha ,\beta ,\theta \right)\right]=n\ln\left(2\right)+n\ln\left(\alpha \right)+n\ln\left(\beta \right)+n\ln\left(\theta \right)-2\sum_{i=1}^{n}\ln\left(x_{i}\right)\\ \; & +\left(\theta -1\right)\sum_{i=1}^{n}\ln\left(1+x_{i}^{-1}\right)+\alpha \beta \sum_{i=1}^{n}\left[1-{\left(1+x_{i}^{-1}\right)}^{\theta }\right]+\sum_{i=1}^{n}\ln\left\{1-e^{\beta \left[1-{\left(1+x_{i}^{-1}\right)}^{\theta }\right]}\right\}\\ \; & +\left(\alpha -1\right)\sum_{i=1}^{n}\ln\left\{2-e^{\beta \left[1-{\left(1+x_{i}^{-1}\right)}^{\theta }\right]}\right\}. \end{array}$$

The maximum likelihood estimates (MLEs) of α, β, and θ, say $\hat{\alpha }$, $\hat{\beta }$, and $\hat{\theta }$, respectively, are defined such that $L\left(\hat{\alpha },\hat{\beta },\hat{\theta }\right)=\max_{\left(\alpha ,\beta ,\theta \right)\in {\left(0,+\infty \right)}^{3}}L\left(\alpha ,\beta ,\theta \right)$ or $\ell \left(\hat{\alpha },\hat{\beta },\hat{\theta }\right)=\max_{\left(\alpha ,\beta ,\theta \right)\in {\left(0,+\infty \right)}^{3}}\ell \left(\alpha ,\beta ,\theta \right)$. Let us work with the function ℓ(α,β,θ) for the sake of simplicity. Since ℓ(α,β,θ) is differentiable with respect to α,β, and θ, the MLEs can obtained by solving the non-linear equations defined by the first partial derivatives of ℓ(α,β,θ) with respect to α,β, and θ equal to 0, with

$$\begin{array}{c} \frac{\partial \ell (\alpha ,\beta ,\theta )}{\partial \alpha }=\frac{n}{\alpha }+\beta \sum_{i=1}^{n}\left[1-{\left(1+x_{i}^{-1}\right)}^{\theta }\right]+\sum_{i=1}^{n}\ln\left\{2-e^{\beta \left[1-{\left(1+x_{i}^{-1}\right)}^{\theta }\right]}\right\}, \end{array}$$

$$\begin{array}{cc} \frac{\partial \ell (\alpha ,\beta ,\theta )}{\partial \beta } & =\frac{n}{\beta }+\alpha \sum_{i=1}^{n}\left[1-{\left(1+x_{i}^{-1}\right)}^{\theta }\right]-\sum_{i=1}^{n}\frac{\left[1-{\left(1+x_{i}^{-1}\right)}^{\theta }\right]e^{\beta \left[1-{\left(1+x_{i}^{-1}\right)}^{\theta }\right]}}{1-e^{\beta \left[1-{\left(1+x_{i}^{-1}\right)}^{\theta }\right]}}\\ \; & -\left(\alpha -1\right)\sum_{i=1}^{n}\frac{\left[1-{\left(1+x_{i}^{-1}\right)}^{\theta }\right]e^{\beta \left[1-{\left(1+x_{i}^{-1}\right)}^{\theta }\right]}}{2-e^{\beta \left[1-{\left(1+x_{i}^{-1}\right)}^{\theta }\right]}} \end{array}$$

and

$$\begin{array}{cc} \frac{\partial \ell (\alpha ,\beta ,\theta )}{\partial \theta } & =\frac{n}{\theta }+\sum_{i=1}^{n}\ln\left(1+x_{i}^{-1}\right)-\alpha \beta \sum_{i=1}^{n}{\left(1+x_{i}^{-1}\right)}^{\theta }\ln\left(1+x_{i}^{-1}\right)\\ \; & +\beta \sum_{i=1}^{n}\frac{{\left(1+x_{i}^{-1}\right)}^{\theta }\ln\left(1+x_{i}^{-1}\right)e^{\beta \left[1-{\left(1+x_{i}^{-1}\right)}^{\theta }\right]}}{1-e^{\beta \left[1-{\left(1+x_{i}^{-1}\right)}^{\theta }\right]}}\\ \; & +\beta \left(\alpha -1\right)\sum_{i=1}^{n}\frac{{\left(1+x_{i}^{-1}\right)}^{\theta }\ln\left(1+x_{i}^{-1}\right)e^{\beta \left[1-{\left(1+x_{i}^{-1}\right)}^{\theta }\right]}}{2-e^{\beta \left[1-{\left(1+x_{i}^{-1}\right)}^{\theta }\right]}}. \end{array}$$

The complexity of these expressions do not allow us to provide closed-forms for the MLEs. However, several numerical solutions exist to maximize ℓ(α,β,θ) based on Newton–Raphson algorithms, one of which is employed in this study.

The corresponding Fisher information matrix we observed is given by

$$J\left(\alpha ,\beta ,\theta \right)=-\left(\begin{array}{ccc} \frac{\partial^{2}\ell \left(\alpha ,\beta ,\theta \right)}{\partial \alpha^{2}} & \frac{\partial^{2}\ell \left(\alpha ,\beta ,\theta \right)}{\partial \alpha \partial \beta } & \frac{\partial^{2}\ell \left(\alpha ,\beta ,\theta \right)}{\partial \alpha \partial \theta }\\ \frac{\partial^{2}\ell \left(\alpha ,\beta ,\theta \right)}{\partial \beta \partial \alpha } & \frac{\partial^{2}\ell \left(\alpha ,\beta ,\theta \right)}{\partial \beta^{2}} & \frac{\partial^{2}\ell \left(\alpha ,\beta ,\theta \right)}{\partial \beta \partial \theta }\\ \frac{\partial^{2}\ell \left(\alpha ,\beta ,\theta \right)}{\partial \theta \partial \alpha } & \frac{\partial^{2}\ell \left(\alpha ,\beta ,\theta \right)}{\partial \theta \partial \beta } & \frac{\partial^{2}\ell \left(\alpha ,\beta ,\theta \right)}{\partial \theta^{2}} \end{array}\right).$$

(the elements of J(α,β,θ) are upon request from the authors). When *n* is large, the distribution of the subjacent random vector behind $\left(\hat{\alpha },\hat{\beta },\hat{\theta }\right)$ can be approximated by a three dimensional normal distribution with mean vector (α,β,θ) and covariance matrix $J{\left(\hat{\alpha },\hat{\beta },\hat{\theta }\right)}^{-1}$. By denoting $v_{\hat{\alpha }}$, $v_{\hat{\beta }}$ and $v_{\hat{\theta }}$, the diagonal elements of this matrix, we are able to construct asymptotic confidence intervals for α, β, and θ. Indeed, with the adopted notations, the asymptotic (equitailed) confidence intervals (CIs) of α, β, and θ at the level 100(1−γ)% are given by, respectively,

$$CI_{\alpha }=\left[\hat{\alpha }-z_{\gamma /2}\sqrt{v_{\hat{\alpha }}},\hat{\alpha }+z_{\gamma /2}\sqrt{v_{\hat{\alpha }}}\right],\;CI_{\beta }=\left[\hat{\beta }-z_{\gamma /2}\sqrt{v_{\hat{\beta }}},\hat{\beta }+z_{\gamma /2}\sqrt{v_{\hat{\beta }}}\right]$$

and

$$CI_{\theta }=\left[\hat{\theta }-z_{\gamma /2}\sqrt{v_{\hat{\theta }}},\hat{\theta }+z_{\gamma /2}\sqrt{v_{\hat{\theta }}}\right],$$

where $z_{\gamma /2}$ is the upper γ/2-th percentile of the normal distribution N(0,1). For practical purposes, if lower bounds of these intervals are negative, we can put it at 0, since all the parameters are supposed to be positive. All the technical details can be found in [33].

### 4.2. Numerical Results

Here, we provide a simulation study to show the nice behavior of the MLEs for the NPTLILx model presented in the subsection above. First of all, let us mention that a random sample from *X* can be obtained by the use of the qf: for any random sample of size *n* from the uniform distribution U(0,1), say $u_{1},\ldots ,u_{n}$, the corresponding random sample of size *n* of *X* is given by $x_{1},\ldots ,x_{n}$ with $x_{i}=Q\left(u_{i};\alpha ,\beta ,\theta \right)$.

From *N* random samples of *X*, let ϵ be either α, β, or θ and ${\hat{\epsilon }}_{i}$ be the MLE of ϵ constructed from the *i*-th sample. Then, we define the (mean) MLE, bias, and mean square error (MSE) by, respectively,

$${\hat{MLE}}_{\epsilon }\left(n\right)=\frac{1}{N}\sum_{i=1}^{N}{\hat{\epsilon }}_{i},\;{\hat{Bias}}_{\epsilon }\left(n\right)={\hat{MLE}}_{\epsilon }\left(n\right)-\epsilon ,\;{\hat{MSE}}_{\epsilon }\left(n\right)=\frac{1}{N}\sum_{i=1}^{N}{\left({\hat{\epsilon }}_{i}-\epsilon \right)}^{2}.$$

Additionally, the asymptotic (mean) confidence intervals of α, β, and θ at the level 100(1−γ)% can be determined. We define the (mean) lower bounds (LBs), (mean) upper bounds (UBs), and (mean) average length (ALs) by, respectively,

$${\hat{LB}}_{\epsilon }\left(n\right)={\hat{MLE}}_{\epsilon }\left(n\right)-z_{\gamma /2}{\hat{V}}_{\epsilon }\left(n\right),\;{\hat{UP}}_{\epsilon }\left(n\right)={\hat{MLE}}_{\epsilon }\left(n\right)+z_{\gamma /2}{\hat{V}}_{\epsilon }\left(n\right),\;{\hat{AL}}_{\epsilon }\left(n\right)=2z_{\gamma /2}{\hat{V}}_{\epsilon }\left(n\right),$$

where ${\hat{V}}_{\epsilon }\left(n\right)=\left(1/N\right)\sum_{i=1}^{N}\sqrt{v_{{\hat{\epsilon }}_{i}}}$. For the purposes of this study, we consider the levels 90% and 95%, so $z_{0.05}=1.644854$ and $z_{0.025}=1.959964$, respectively. The software Mathematica 9 was employed.

Our simulation study was based on the the following plan.

- N=1000 random samples of size n=100, 200, 300, and 1000 are to be generated from *X*.
- Values of the true parameters (α,β,θ) are taken as, in order, (0.5,0.1,0.5), (1.5,0.5,0.5), and (1.8,0.4,1.2).
- The MLEs, MSEs, biases, LBs, UBs, and ALs for the selected values of the parameters are to be calculated.

Numerical outcomes are listed in Table 4, Table 5 and Table 6.

From Table 4, Table 5 and Table 6, we can see that, when *n* increases, biases, MSEs, and ALs decrease. This observation is consistent with the well-known convergence properties of the MLEs.

## 5. Data Analysis

In this section, we prove the flexibility of the NPTLILx model by analyzing two practical datasets. The fits of the NPTLILx model are compared to the competitive models listed in Table 7. The common point of all of them is the use the inverse Lomax distribution as the baseline distribution.

Except the former inverse Lomax distribution, the considered models possess three or four parameters. The comparison of these models was performed by using the following well-known statistical benchmarks: CVM (Cramér–von Mises); AD (Anderson–Darling); KS (Kolmogorov–Smirnov) statistic with the corresponding *p*-value, minus log-likelihood $\left(-\hat{\ell }\right)$; AIC (Akaike information criterion); CAIC (corrected Akaike information criterion); BIC (Bayesian information criterion); and HQIC (Hannan–Quinn information criterion). For the CVM, AD, KS, $\left(-\hat{\ell }\right)$, AIC, CAIC, BIC, and HQIC, the smaller the value is, the better the fit to the data. Additionally, the higher the *p*-values of the KS test are, the better the fit to the data. All these measures were computed by using the R software.

Dataset I: The first data refer to [23]. It consists of 40 observations of the active repair times for airborne communication transceiver. The unit is the hour. The data are: 0.50, 0.60, 0.60, 0.70, 0.70, 0.70, 0.80, 0.80, 1.00, 1.00, 1.00, 1.00, 1.10, 1.30, 1.50, 1.50, 1.50, 1.50, 2.00, 2.00, 2.20, 2.50, 2.70, 3.00, 3.00, 3.30, 4.00, 4.00, 4.50, 4.70, 5.00, 5.40, 5.40, 7.00, 7.50, 8.80, 9.00, 10.20, 22.00, 24.50.

A basic statistical description of the data gives: n=40, mean =4.01, standard deviation =5.17, median =2.1, skewness =2.62, and kurtosis =7.02. One can notice that the data are skewed to the right with a high kurtosis.

Dataset II: Next, we use the actual taxes dataset as described in [24]. The data consist of the monthly actual taxes revenue in Egypt from January 2006 to November 2010. The unit is the 1000 million Egyptian pounds. The data are: 5.9, 20.4, 14.9, 16.2, 17.2, 7.8, 6.1, 9.2, 10.2, 9.6, 13.3, 8.5, 21.6, 18.5,5.1,6.7, 17, 8.6, 9.7, 39.2, 35.7, 15.7, 9.7, 10, 4.1, 36, 8.5, 8, 9.2, 26.2, 21.9, 16.7, 21.3, 35.4, 14.3, 8.5, 10.6, 19.1, 20.5, 7.1, 7.7, 18.1, 16.5, 11.9, 7, 8.6, 12.5, 10.3, 11.2, 6.1, 8.4, 11, 11.6, 11.9, 5.2, 6.8, 8.9, 7.1, 10.8.

A basic statistical description of the data gives: n=59, mean =13.49, standard deviation =8.05, median =10.6, skewness =1.57, and kurtosis =2.08. Thus, these data are skewed to the right with a moderate kurtosis.

**Figure 3:**
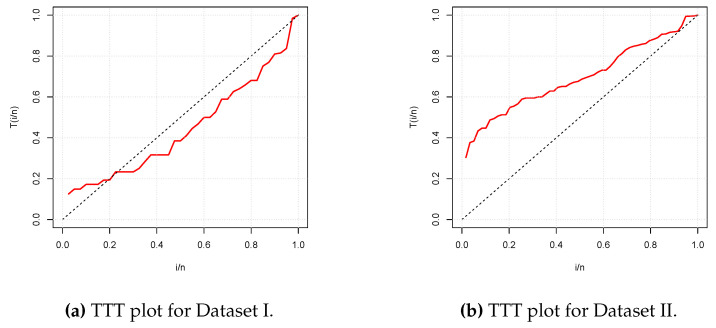
Total test time (TTT) plots for Datasets I and II, respectively.

The graphical and numerical analyses of these two datasets are as follows. Figure 3 presents the total test time (TTT) plots of the two datasets. The first plot shows a convex curve, indicating that a decreasing hrf for the fitting model is appropriate for Data set I, whereas the second plot shows a concave curve, indicating that an increasing hrf for the fitting model is appropriate for Data set II. These cases are covered by the NPTLILx model, as shown in Figure 2.

**Figure 4:**
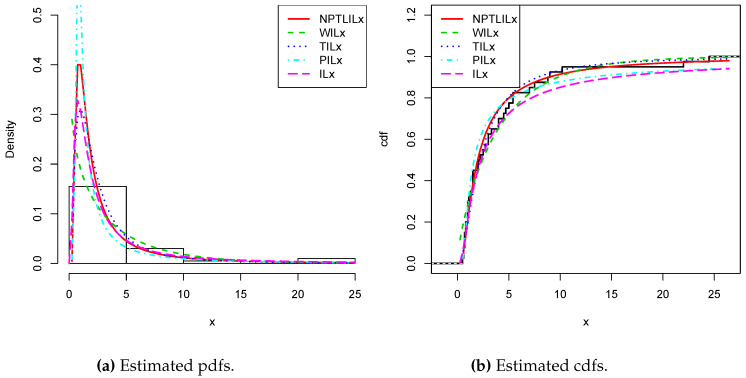
Plots for the estimated pdfs and cdfs for Dataset I.

**Figure 5:**
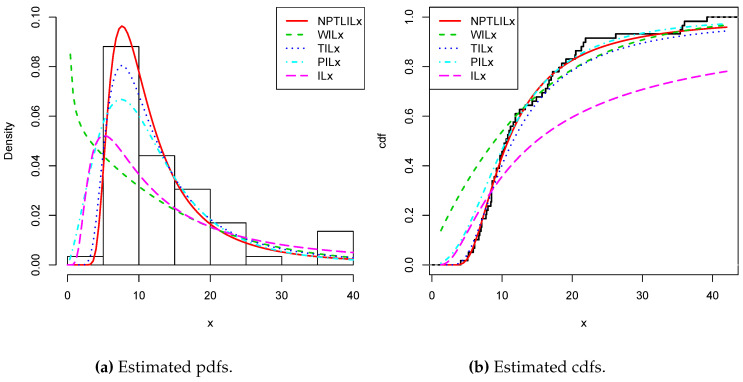
Plots for the estimated pdfs and cdfs for Dataset II.

**Figure 6:**
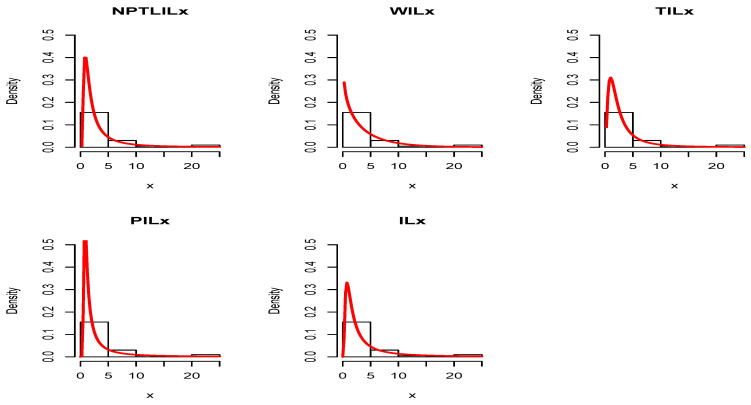
Plots of the pdfs estimated for Dataset I.

**Figure 7:**
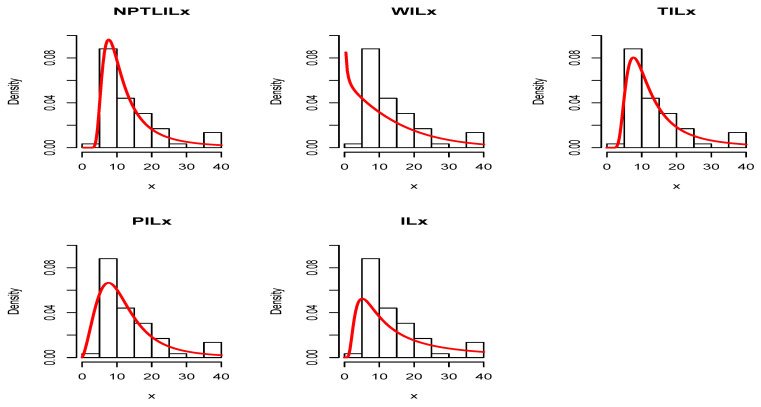
Plots of the pdfs estimated for Dataset II.

**Figure 8:**
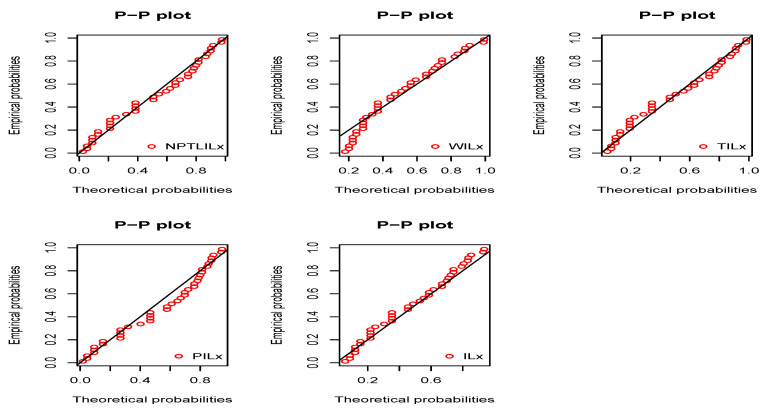
Probability–probability (PP) plots of considered models for Dataset I.

**Figure 9:**
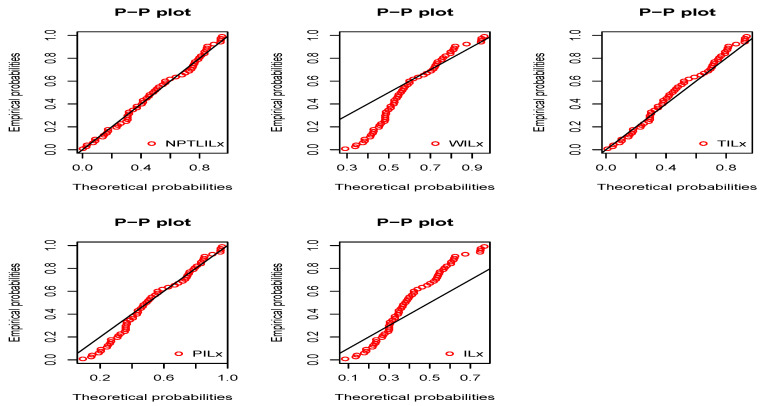
PP plots of considered models for Dataset II.

Table 8 and Table 9 present the CVM, AD, KS, and the related *p*-value, and the MLEs of the models’ parameters for Datasets I and II, respectively. The obtained *p*-values indicate that the NPTLILx model is the best. Table 10 and Table 11 communicate the $-\hat{\ell }$, AIC, BIC, CAIC, BIC, and HQIC of the models for Datasets I and II, respectively. Since the smallest values are obtained for the NPTLILx model, it can be considered the best with these criteria. The estimated pdfs and cdfs for the considered models are displayed in Figure 4 and Figure 5 for Datasets I and II, respectively. The plots of the estimated pdfs are visually refined via an individual treatment in Figure 6 and Figure 7. In order to give another point of view, we illustrate the adequateness of the models via the use of probability–probability (PP) plots in Figure 8 and Figure 9, for Datasets I and II, respectively. In particular, for Dataset II, in view of the perfect adjustment of the scatter plot by the PP line, it is clear that the NPTLILx model provides a better fit in comparison to the other models. To resume, the NPTLILx model reveals itself to be the more appropriate model for the two datasets, illustrating its applicability in a concrete setting.

**Figure 10:**
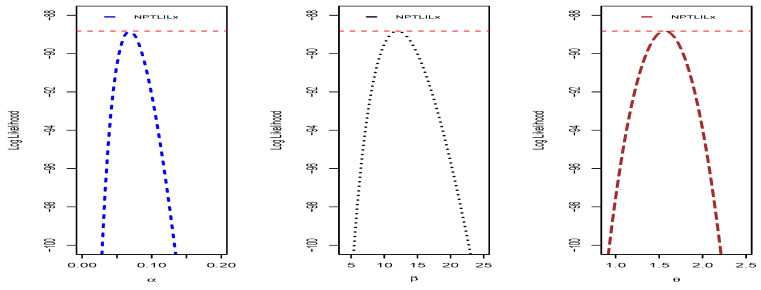
Profiles of the log-likelihood function of the NPTLILx model for Dataset I.

**Figure 11:**
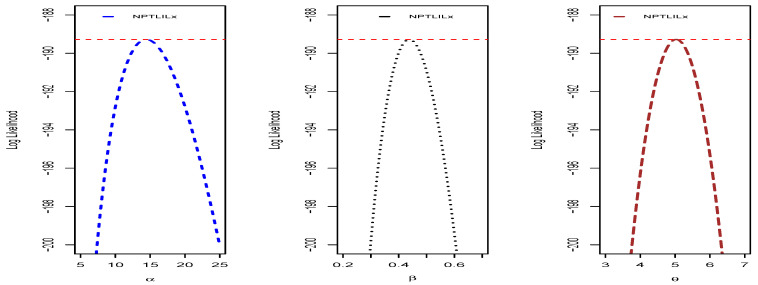
Profiles of the log-likelihood function of the NPTLILx model for Dataset II.

We end this section by providing some additional graphical and numerical elements on the NPTLILx model, related to the quantities presented in Section 4.1. To illustrate the uniqueness of the MLEs of α, β and θ, the profiles of the log-likelihood function are proposed in Figure 10 and Figure 11 for Datasets I and II, respectively. The Fisher information matrices of the NPTLILx model taken at the MLEs for Datasets I and II are, respectively, given by

$$J_{I}=\left(\begin{array}{ccc} 0.0080 & -0.0354 & -0.0018\\ -0.0354 & 5.8404 & 0.0216\\ -0.0018 & 0.0216 & 0.4128 \end{array}\right),\;J_{II}=\left(\begin{array}{ccc} 1.0770 & -1.2877 & -0.0627\\ -1.2877 & 1.2327 & 0.0654\\ -0.0627 & 0.0654 & 0.0590 \end{array}\right).$$

Then, the confidence intervals for α, β, and θ at the levels 90% and 95% are provided in Table 12.

## 6. Conclusion and Perspectives

In this paper, we introduced and studied a new general family of distributions, called the NPTL-G family, based on the so-called power Topp–Leone-G and inverse exponential-G families. Various mathematical properties were presented, including stochastic ordering, quantile function and related measures, general moments and related measures, and the Shannon entropy, with discussions. Then, we payed special attention to a member of the family defined with the inverse Lomax distribution, called the NPTLILx distribution. The estimation of the unknown model parameters was done with the maximum likelihood method, with numerical guarantees on their behavior via a simulation study. The applicability of the NPTLILx model was then illustrated by the consideration of two practical datasets. It was then proven that the NPTLILx model is a serious alternative to other models, also using the inverse Lomax distribution as the baseline. Future work will include the constructions of various regression models, Bayesian estimation of the parameters, and analyses of new datasets. Thanks to its numerous qualities, we believe that the NPTL-G family can be helpful for the practitioner, for statistical analyses beyond the scope of this paper.

Among the interesting perspectives of work, one could investigate the confidence bounds and supersaturation properties of the cdfs of the members of the NPTL-G family, which are useful for choosing an appropriate model for given data, following the spirit of [38,39,40,41,42]. All these aspects need further investigations that we leave for future works.

## Figures and Tables

**Table 1:** The values of $Q_{1}$, *M*, $Q_{3}$, *S*, and *K* of the NPTLILx distribution for some parameter values.

(α,β,θ)	$Q_{1}$	*M*	$Q_{3}$	S	K
(0.5,0.5,0.5)	0.0162	0.0413	0.1108	0.4703	1.8976
(1.5,0.5,0.5)	0.0667	0.1377	0.3000	0.3917	1.7017
(2.5,0.5,0.5)	0.1150	0.2200	0.4464	0.3664	1.6542
(3.5,0.5,0.5)	0.1594	0.2922	0.5701	0.3533	1.6323
(5.0,0.5,0.5)	0.2200	0.3877	0.7298	0.3421	1.6151
(5.0,1.0,0.5)	0.5513	0.9168	1.6330	0.3244	1.5894
(5.0,2.0,0.5)	1.2624	2.0174	3.4724	0.3167	1.5807
(5.0,3.0,0.5)	1.9896	3.1308	5.3207	0.3148	1.5788
(5.0,5.0,0.5)	3.4563	5.3666	9.0231	0.3137	1.5778
(5.0,5.0,1.0)	7.3808	11.2118	18.5331	0.3130	1.5772
(5.0,5.0,2.0)	15.2458	22.9128	37.5597	0.3128	1.5771
(5.0,5.0,3.0)	23.1143	34.6163	56.5877	0.3128	1.5771
(5.0,5.0,5.0)	38.8534	58.0246	94.6445	0.3128	1.5771

**Table 2:** The values for the first four incomplete moments of the NPTLILx distribution; i.e., $\mu_{s}^{{}^{\prime }}\left(y\right)$ with s=1,2,3,4, with y=1000, for some parameter values.

(α,β,θ)	$\mu_{1}^{{}^{\prime }}\left(1000\right)$	$\mu_{2}^{{}^{\prime }}\left(1000\right)$	$\mu_{3}^{{}^{\prime }}\left(1000\right)$	$\mu_{4}^{{}^{\prime }}\left(1000\right)$
(0.5,0.5,0.5)	0.1330	0.4114	62.0150	31180.1500
(1.0,0.5,0.5)	0.2289	0.8177	124.0269	62360.2800
(3.0,0.5,0.5)	0.5037	2.4083	372.0441	187080.6
(0.5,1.0,0.5)	0.3216	1.5544	247.4997	124628
(0.5,3.0,0.5)	1.1648	12.2536	2209.2820	1118342
(0.5,0.5,1.0)	0.4499	1.6965	248.6065	124813.2
(0.5,0.5,3.0)	3.6376	41.6806	6308.0930	3138854
(2.0,2.0,0.5)	1.8826	22.1519	3941.2640	1991066
(2.0,2.0,2.0)	8.7637	288.4201	62161.91	31710832
(2.0,2.0,15)	65.7921	9473.519	3149044	1707182402
(2.0,2.0,30)	125.1442	28393.86	11331002	6455199311
(2.0,5.0,20)	180.9897	55135.34	25604726	15505315569
(5.0,5.0,20)	268.5182	106564.8	56092507	35760970261
(10,10,10)	337.2701	160356.5	93263392	62533636278

**Table 3:** The values of the Shannon entropy of the NPTLILx distribution for some parameter values.

α	β	θ	η
0.5	0.5	0.5	−1.3743
1.0	0.5	0.5	−0.7246
2.0	0.5	0.5	−0.1757
3.0	0.5	0.5	0.1094
5.0	0.5	0.5	0.4405
10	0.5	0.5	0.8528
10	1.0	0.5	1.5895
10	2.0	0.5	2.2907
10	5.0	0.5	3.1614
10	8	0.5	3.5497
0.5	0.5	0.1	−12.8618
5.0	0.5	0.1	−3.9876
5.0	5.0	0.1	1.1590

**Table 4:** Maximum likelihoods (MLEs), biases, MSEs, LBs, UBs, and ALs of the NPTLILx model for (α=0.5,β=0.1,θ=0.5).

*n*	Par.	ML	Bias	MSE	90%			95%		
					LB	UB	AL	LB	UB	AL
100	α	0.290	−0.210	0.105	−0.528	1.109	1.637	−0.685	1.266	1.951
	β	0.163	0.063	0.016	−0.090	0.417	0.508	−0.139	0.466	0.605
	θ	0.572	0.072	0.012	0.364	0.780	0.416	0.324	0.820	0.496
200	α	0.304	−0.196	0.103	−0.329	0.937	1.266	−0.450	1.058	1.508
	β	0.146	0.046	0.008	−0.031	0.324	0.354	−0.065	0.358	0.422
	θ	0.567	0.067	0.011	0.386	0.749	0.363	0.351	0.784	0.433
300	α	0.340	−0.160	0.084	−0.264	0.944	1.207	−0.379	1.059	1.438
	β	0.137	0.037	0.006	0.021	0.254	0.233	−0.001	0.276	0.277
	θ	0.548	0.048	0.007	0.414	0.683	0.269	0.388	0.709	0.321
1000	α	0.342	−0.158	0.074	−0.064	0.747	0.811	−0.141	0.825	0.966
	β	0.124	0.024	0.002	0.047	0.200	0.153	0.033	0.215	0.182
	θ	0.545	0.045	0.005	0.435	0.655	0.220	0.414	0.676	0.263

**Table 5:** MLEs, biases, MSEs, LBs, UBs, and ALs of the NPTLILx model for (α=1.5,β=0.5,θ=0.5).

*n*	Par.	ML	Bias	MSE	90%			95%		
					LB	UB	AL	LB	UB	AL
100	α	2.042	0.542	5.123	−211.872	215.955	427.827	−252.834	256.918	509.752
	β	0.373	−0.127	0.055	−8.964	9.711	18.674	−10.752	11.499	22.250
	θ	0.618	0.118	0.060	−30.579	31.814	62.393	−36.553	37.788	74.341
200	α	1.843	0.343	1.449	−81.290	84.975	166.265	−97.209	100.894	198.103
	β	0.442	−0.058	0.015	−4.239	5.124	9.363	−5.136	6.021	11.156
	θ	0.547	0.047	0.019	−10.112	11.206	21.317	−12.153	13.247	25.400
300	α	1.667	0.167	0.874	−45.858	49.192	95.050	−54.959	58.293	113.252
	β	0.458	−0.042	0.012	−2.019	2.935	4.954	−2.493	3.410	5.903
	θ	0.542	0.042	0.013	−5.733	6.818	12.550	−6.934	8.019	14.953
1000	α	1.358	−0.142	0.460	−2.292	5.008	7.300	−2.991	5.707	8.698
	β	0.534	0.034	0.002	0.184	0.884	0.700	0.117	0.951	0.834
	θ	0.534	0.034	0.010	0.279	0.788	0.509	0.230	0.837	0.607

**Table 6:** MLEs, biases, MSEs, LBs, UBs, and ALs of the NPTLILx model for (α=1.8,β=0.4,θ=1.2).

*n*	Par.	ML	Bias	MSE	90%			95%		
					LB	UB	AL	LB	UB	AL
100	α	2.843	1.043	1.889	−1324.960	1330.640	2655.600	−1579.220	1584.900	3164.120
	β	0.639	0.239	0.675	−94.183	95.462	189.645	−112.341	113.619	225.960
	θ	1.573	0.373	0.660	−252.661	255.807	508.468	−301.344	304.490	605.834
200	α	1.095	−0.705	0.523	−359.428	361.618	721.046	−428.464	430.654	859.119
	β	0.331	−0.069	0.014	−27.830	28.493	56.324	−33.223	33.886	67.109
	θ	1.571	0.371	0.183	−109.805	112.947	222.752	−131.132	134.274	265.406
300	α	1.150	−0.650	0.519	−306.474	309.685	616.159	−365.468	368.679	734.147
	β	0.421	0.021	0.004	−18.962	19.804	38.766	−22.674	23.515	46.189
	θ	1.390	0.190	0.165	−92.650	95.370	188.020	−110.652	113.372	224.024
1000	α	1.260	−0.540	0.496	−138.760	141.281	280.040	−165.572	168.093	333.665
	β	0.387	−0.013	0.001	−9.334	10.007	19.341	−11.186	11.859	23.044
	θ	1.361	0.161	0.150	−45.112	48.295	93.407	−54.055	57.238	111.293

**Table 7:** The competitive models considered.

Distribution	Reference
Inverse Lomax (ILx)	[34]
Inverse Power Lomax (PILx)	[35]
Topp–Leone Inverse Lomax (TILx)	[36]
Weibull Inverse Lomax (WILx)	[37]

**Table 8:** Goodness-of-fit measures, MLEs, and SEs for Dataset I.

Model	CVM	AD	KS	*p*-Value	MLEs with SEs (in Parentheses)			
NPTLILx	0.0550	0.3462	0.0943	0.8683	α	β	θ	
					0.0682	11.9902	1.5651	
					(0.0897)	(2.4167)	(0.6425)	
WILx	0.1522	1.0852	0.1784	0.1566	*a*	*b*	λ	β
					0.0026	0.8867	0.0185	0.2581
					(0.0006)	(0.1938)	(0.0285)	(0.7650)
TILx	0.0685	0.4578	0.1108	0.7100	α	β	λ	
					37.8324	2.9879	0.1676	
					(9.7598)	(1.9941)	(0.2682)	
PILx	0.1079	0.6582	0.1272	0.5369	α	β	λ	
					0.1130	6.8594	0.0571	
					(0.1242)	(6.4470)	(0.2480)	
ILx	0.0632	0.4065	0.0981	0.8355	α	β		
					0.2003	8.2426		
					(0.1372)	(5.1671)		

**Table 9:** Goodness-of-fit measures, MLEs, and SEs for Dataset II.

Model	CVM	AD	KS	*p*-Value	MLEs with SEs (in Parentheses)			
NPTLILx	0.0357	0.2698	0.0615	0.9786	α	β	θ	
					14.4361	0.4378	5.0301	
					(1.0378)	(1.1103)	(0.2431)	
WILx	0.2363	1.4829	0.3248	$7.79\times 10^{-6}$	*a*	*b*	λ	β
					0.0021	1.1404	0.0172	3.9985
					(0.0002)	(0.1202)	(0.0064)	(3.1371)
TILx	0.0398	0.2701	0.0998	0.5988	α	β	λ	
					50.4579	0.0908	15.8617	
					(6.7245)	(0.2094)	(3.9080)	
PILx	0.1133	0.6440	0.1447	0.1689	α	β	λ	
					1.1545	2.3262	300.7315	
					(0.4472)	(0.2880)	(121.3061)	
ILx	0.0529	0.3075	0.2928	$8.06\times 10^{-5}$	α	β		
					0.1464	71.1473		
					(0.2569)	(23.8010)		

**Table 10:** The values of $-\hat{\ell }$, AIC, and KS with its *p*-value for Dataset I.

Model	$-\hat{\ell }$	AIC	CAIC	BIC	HQIC
NPTLILx	88.8229	183.6459	184.3125	188.7125	185.4778
WILx	98.2937	204.5874	205.7303	211.3429	207.0300
TILx	90.5459	187.0919	187.7586	192.1586	188.9239
PILx	90.5908	187.1817	187.8484	192.2483	189.0136
ILx	91.3612	186.7226	187.0469	190.1003	187.9439

**Table 11:** The values of $-\hat{\ell }$, AIC, and KS with its *p*-value for Dataset II.

Model	$-\hat{\ell }$	AIC	CAIC	BIC	HQIC
NPTLILx	189.2811	384.5622	384.9985	390.7948	386.9951
WILx	219.6212	447.2424	447.9832	455.5526	450.4864
TILx	190.3769	386.7538	387.1902	392.9864	389.1868
PILx	195.1056	396.2113	396.6476	402.4439	398.6442
ILx	211.6436	427.2872	427.5015	431.4422	428.9091

**Table 12:** Confidence intervals for the parameters of the NPTLILx model for Datasets I and II, respectively.

CI	α	β	θ
90%	[0 0.2157]	[8.0147 15.9656]	[0.5081 2.6220]
95%	[0 0.2440]	[7.2534 16.7269]	[0.3058 2.8244]
**CI**	α	β	θ
90%	[12.7289 16.1432]	[0 2.2642]	[4.6302 5.4300]
95%	[12.4020 16.4701]	[0 2.6139]	[4.5536 5.5065]
